# Heavy metals' data in soils for agricultural activities

**DOI:** 10.1016/j.dib.2018.04.115

**Published:** 2018-05-05

**Authors:** T.A. Adagunodo, L.A. Sunmonu, M.E. Emetere

**Affiliations:** a Department of Physics, Covenant University, Ota, Nigeria; b Department of Pure and Applied Physics, Ladoke Akintola University of Technology, Ogbomoso, Nigeria

**Keywords:** Agricultural soils, Heavy metals, Contamination, Environment, Soil screening, Geostatistics

## Abstract

In this article, the heavy metals in soils for agricultural activities were analyzed statistically. Ten (10) soil samples were randomly taken across the agricultural zones in Odo-Oba, southwestern Nigeria. Ten (10) metals; namely: copper (Cu), lead (Pb), chromium (Cr), arsenic (As), zinc (Zn), cadmium (Cd), nickel (Ni), antimony (Sb), cobalt (Co) and vanadium (V) were determined and compared with the guideline values. When the values were compared with the international standard, none of the heavy metals in the study area exceeded the threshold limit. However, the maximum range of the samples showed that Cr and V exceeded the permissible limit which could be associated with ecological risk. The data can reveal the distributions of heavy metals in the agricultural topsoil of Odo-Oba, and can be used to estimate the risks associated with the consumption of crops grown on such soils.

**Specifications Table**

**Table:** 

Subject area	*Earth Planetary Science*
More specific subject area	*Environmental Geophysics, Geochemistry, Soil Science*
Type of data	*Table and figure*
How data was acquired	*Inductively Coupled Plasma Mass Spectrometry*
Data format	*Raw and analyzed*
Experimental factors	*Agricultural soils were randomly taken for heavy metal analysis*
Experimental features	*The ten metals as stated in the abstract were analyzed statistically and compared with the guideline values*
Data source location	*Odo-Oba, Southwestern Nigeria*
Data accessibility	*All the data are in this article*

**Value of the data**

• The data would give insight on the concentrations of heavy metals in the agricultural soils of the study area.
• The data from this study could be used to study the relationships between the subsurface heavy metals and the rate of germination as well as productivity of the crops in the study area.
• The study could be used to predict appropriate crops that could easily survive on the agricultural soils.
• The data could be used for soil screening and to measure the food security strength in the environment.

## 1 Data

The data contains the geoexploration and geostatistical analysis of heavy metals in agricultural soils of Odo-Oba, southwestern Nigeria. Ten (10) samples were randomly collected for heavy metal analysis. Heavy metals are the metallic elements which exhibit relatively high density when compared with the density of water. The toxicity of heavy metals ranged from the route of exposure to the doses received [1]. In this article, ten (10) metals which are significant to the public health have been analyzed. The variables are: copper (Cu), lead (Pb), chromium (Cr), arsenic (As), zinc (Zn), cadmium (Cd), nickel (Ni), antimony (Sb), cobalt (Co) and vanadium (V). The results of the heavy metals from the study area are presented in Table 1. The data were compared with the international regulatory standard [2], which is presented in Table 2. The standards in Table 2 are grouped under threshold and permissible limits. These limits have been applied across the globe to measure the heavy metal contents in agricultural soils [3]. The threshold limit is used to checkmate the minimum toxicity in all soils environment. The permissible limit is applicable to the agricultural soils. If the values of the heavy metals exceed the permissible limit, such soil is regarded as contaminated soils for agricultural activities [1], [2], [4], [5]. It is either associated with health risk (hr) or ecological risk (er). However, descriptive analyses were further used to explore the heavy metals’ results, which are presented in Table 3a, Table 3b.

**Table 1:** Heavy metals in Odo-Oba.

Samples	Variables (mg kg^−1^)									
	Cu	Pb	Cr	As	Zn	Cd	Ni	Sb	Co	V
Soil1	6.43	25.88	43.00	2.40	29.40	0.02	10.20	0.11	6.80	34.00
Soil2	5.26	20.89	31.00	1.70	29.00	0.02	9.30	0.09	6.80	24.00
Soil3	5.32	22.21	23.00	2.20	24.10	0.03	7.90	0.06	6.80	27.00
Soil4	10.06	30.90	44.00	2.50	61.30	0.05	15.20	0.15	13.00	40.00
Soil5	5.69	19.13	26.00	1.60	25.80	0.04	9.40	0.27	6.80	27.00
Soil6	3.91	18.99	24.00	1.70	31.90	0.03	8.20	0.16	6.30	22.00
Soil7	7.01	43.89	69.00	2.00	24.90	0.03	18.10	0.07	11.90	45.00
Soil8	20.69	40.15	341.00	3.50	31.00	0.06	31.80	0.16	17.90	124.00
Soil9	19.51	31.63	125.00	3.70	31.50	0.02	26.50	0.14	19.10	89.00
Soil10	7.51	30.07	86.00	2.70	22.80	0.03	15.80	0.07	10.50	45.00

**Table 2:** Threshold and permissible limits for heavy metals in soils.

Variables	Threshold limit (mg kg^−1^) [1], [2]	Permissible limit (mg kg^−1^) [1], [2]	Present Study (mg kg^−1^)	
			Range	Mean
Cu	100.0	50.0 (er)	3.91–20.69	9.14
Pb	60.0	200.0 (hr)	18.99–43.89	28.37
Cr	100.0	200.0 (er)	23.00–341.00	81.20
As	5.0	50.0 (er)	1.60–3.70	2.40
Zn	200.0	250.0 (er)	22.80–61.30	31.17
Cd	1.0	10.0 (er)	0.02–0.06	0.03
Ni	50.0	100.0 (er)	7.90–31.80	15.24
Sb	2.0	10.0 (hr)	0.06–0.27	0.13
Co	20.0	100.0 (er)	6.30–19.10	10.59
V	100.0	150.0 (er)	22.00–124.00	47.70

Note: The risk associated with higher concentrations greater than the permissible limits are grouped into ecological risk (er) and health risk (hr).

**Table 3a:** Descriptive statistics results for heavy metals (SET A).

Var.	*N*	Mean	SD	SEM	Variance	Sum	Skew	Kurt	USS	CSS	CV	MAD
Cu	10	9.14	6.01	1.91	36.14	91.39	1.49	0.81	1160.44	325.23	0.66	4.57
Pb	10	28.37	8.65	2.73	74.78	283.74	0.67	− 0.55	8723.85	673.02	0.30	6.95
Cr	10	81.20	96.99	30.67	9406.18	812.00	2.56	7.01	150590.0	84655.6	1.19	61.68
As	10	2.40	0.73	0.23	0.54	24.00	0.81	− 0.35	62.42	4.82	0.30	0.56
Zn	10	31.17	11.08	3.50	122.72	311.70	2.66	7.75	10820.21	1104.52	0.36	6.24
Cd	10	0.03	0.01	0.004	1.79 E-4	0.33	1.06	0.46	0.01	0.002	0.41	0.01
Ni	10	15.24	8.22	2.60	67.53	152.40	1.18	0.44	2930.32	607.74	0.54	6.25
Sb	10	0.13	0.06	0.02	0.004	1.28	1.20	1.88	0.20	0.04	0.49	0.05
Co	10	10.59	4.82	1.52	23.25	105.90	0.90	− 0.58	1330.73	209.25	0.46	3.91
V	10	47.70	33.11	10.47	1096.46	477.00	1.77	2.55	32621.00	9868.10	0.69	23.52

**Table 3b:** Descriptive statistics results for heavy metals (SET B).

Var.	*N*	GM	GSD	Mode	SW	Min	Im	Q1	Median	Q3	Max	IM	IR	Range
Cu	10	7.82	1.74	–	10	3.91	7	5.32	6.72	10.06	20.69	9	4.74	16.78
Pb	10	27.25	1.35	–	10	18.99	7	20.89	27.98	31.63	43.89	8	10.74	24.90
Cr	10	24.05	2.37	–	10	23.00	4	26.00	43.50	86.00	341.0	9	60.0	318.0
As	10	2.31	1.34	1.70	10	1.60	6	1.70	2.30	2.70	3.70	10	1.0	2.10
Zn	10	29.92	1.32	–	10	22.80	11	24.90	29.20	31.50	61.30	5	6.60	38.50
Cd	10	0.03	1.46	0.03	10	0.02	2	0.02	0.03	0.04	0.06	9	0.02	0.04
Ni	10	13.57	1.64	–	10	7.90	4	9.30	12.70	18.10	31.80	9	8.80	23.90
Sb	10	0.12	1.61	0.07	10	0.06	4	0.07	0.13	0.16	0.27	6	0.09	0.21
Co	10	9.71	1.54	6.80	10	6.30	7	6.80	8.65	13.00	19.10	10	6.20	12.80
V	10	40.44	1.76	27.0	10	22.0	7	27.00	37.00	45.00	124.0	9	18.0	102.0

## 2 Experimental design, materials and methods

Exploration of data sets in differs ways have been presented in [6], [7], [8], [9], [10], [11]. Studies on the analysis of soils’ usability for agricultural purposes could be found in [12], [13], [14], [15], [16].

### 2.1 Study area

The data were taken from the agricultural zones in Odo-Oba, southwestern Nigeria. The study area plays a key role in sustaining the food security of Ogbomoso and its environs. The major occupation of the residents in the study area is fishing and farming. Among the crops being cultivated in Odo-Oba are vegetables, tuber crops, leguminous crops and cereals crops [6]. The climatic conditions of the study area are the same as that of Ogbomoso, which have been discussed in [6], [17].

**Fig. 1:**
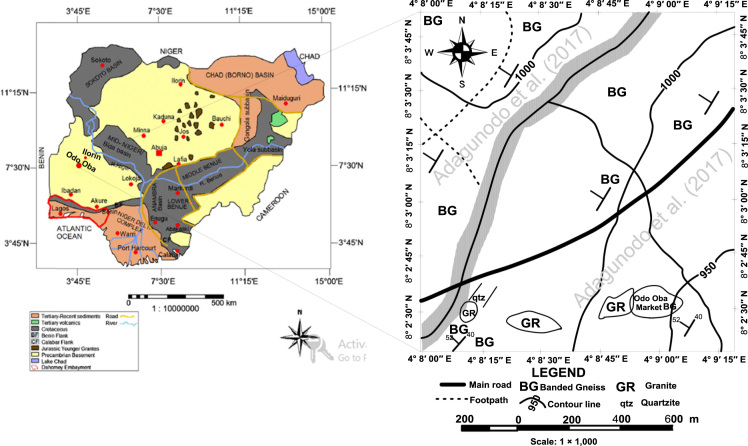
Geology and location of Odo-Oba (modified after [3]).

The geology of Odo-Oba is of Precambrian Basement complex [18], [19], [20], [21], [22], [23], which is an integral part of African igneous and meta-sedimentary rocks [7]. In Nigeria, two geological terrains, namely: Sedimentary Basins [24], [25], [26] and Precambrian Basement complex [27], [28], [29] are divided in equal proportion [30], [31]. The notable rocks in the study area are quartzite, banded gneiss and granites (Fig. 1).

### 2.2 Materials and methods

The samples were randomly collected from ten (10) locations, with the labeling ranging from Soil1 to Soil10. The labeled samples were dried under ambient temperature and sieved in order to remove the unwanted materials within the collected samples. The samples were packaged in plastic sock and moved to Canada for procedural analysis. The heavy metals’ analysis was done in ACME Laboratories using Inductively Coupled Plasma Mass Spectrometry (ICP-MS) technique. The standard procedures were followed during samples’ collection [32], [33] and analysis stages [34].

### 2.3 Statistical analysis

The range of each element was shown in Table 2. None of the mean value exceeded the threshold and the permissible limits. The maximum range of the samples showed that Cr and V exceeded the permissible limit which could be associated with ecological risk in the study area. Table 3a, Table 3b show the comprehensive descriptive statistics of the data. Twenty-five (25) parameters were used to describe the distribution of the heavy metals in Odo-Oba. The results were presented as Table 3a, Table 3b. The population number (*N*), mean, standard deviation (SD), standard error of mean (SEM), variance, sum, skewness (Skew), kurtosis (Kurt), uncorrected sum of squares (USS), corrected sum of squares (CSS), coefficient of variation (CV), mean absolute deviation (MAD), geometric mean (GM), geometric standard deviation (GSD), mode, sum of weights (SW), minimum (Min), index of minimum (Im), 1st quartile (Q1), median, 3rd quartile (Q3), maximum (Max), index of maximum (IM), Interquartile range (IR), and range were presented as the descriptive parameters in the two tables.

Normality tests were further applied to the data sets in order to ensure if the values are modeled from the normal distribution based on the small sample size of the variables. The Lilliefors, Shapiro-Wilk and Kolmogorov-Smirnov normality tests were applied on the data sets. The results are shown in Table 4. In all the three tests, good fitting exist among the variables.

**Table 4:** The normality test results.

Parameters	DF	Shapiro-Wilk		Lilliefors		Kolmogorov-Smirnov	
		Statistic	Prob < *W*	Statistic	Prob > *D*	Statistic	Prob > *D*
Cu	10	0.7461	0.0032	0.3068	0.0083	0.3068	0.2479
Pb	10	0.9093	0.2760	0.1620	0.2000	0.1620	1.0000
Cr	10	0.6426	1.7875E−4	0.2803	0.0251	0.2803	0.3463
As	10	0.8982	0.2094	0.1457	0.2000	0.1457	1.0000
Zn	10	0.6466	1.9950E−4	0.3737	2.8554E−4	0.3737	0.0921
Cd	10	0.8551	0.0668	0.2887	0.0179	0.2887	0.3123
Ni	10	0.8408	0.0451	0.2302	0.1329	0.2302	0.6017
Sb	10	0.8806	0.1325	0.2063	0.2000	0.2063	0.7514
Co	10	0.8120	0.0253	0.2841	0.0216	0.2841	0.3307
V	10	0.7532	0.0039	0.3325	0.0025	0.3325	0.1738

Note: DF is the degree of freedom; at the 0.05, the data was not significantly drawn from a normally distributed population.

**Fig. 2:**
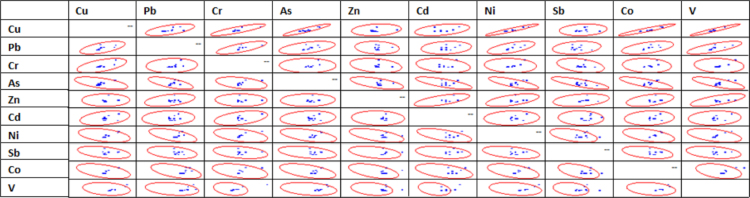
Scatter matrix of heavy metals.

Correlation analyses among the variables were determined in order to visualize the kind of relationships that exist among the analyzed variables using Pearson (Table 5a), Spearman (Table 5b), and Kendall (Table 5c) correlations respectively. The distances between two correlated results were obtained by transforming the results from Table 5a, Table 5b, Table 5c using Eqs. (1), (2), (3). The results of these transformations were presented in Table 6a, Table 6b. The scatter matrix plot of the correlated variables was shown in Fig. 2. It is a statistical tool that enables the estimation of the covariance matrix [8] (Table 6c).

(1) T1=|P–S|

(2) T2=|K–P|

(3) T3=|S–K|

where *T* is the transformation, *P* is the Pearson correlation, *S* is the Spearman correlation, and *K* is the Kendall correlation.

**Table 5a:** Results from Pearson correlation.

Variables	Cu	Pb	Cr	As	Zn	Cd	Ni	Sb	Co	V
Cu	1	0.5912	0.8479	0.9230	0.1854	0.4044	0.9508	0.1553	0.9477	0.9639
Pb		1	0.6364	0.5650	0.0820	0.3637	0.7806	− 0.2828	0.7369	0.6747
Cr			1	0.7456	− 0.0281	0.5948	0.8933	0.0967	0.7546	0.9504
As				1	0.1366	0.2384	0.8702	− 0.0889	0.8892	0.8822
Zn					1	0.4349	0.0940	0.2168	0.2614	0.0371
Cd						1	0.4396	0.4284	0.3796	0.4739
Ni							1	0.0485	0.9597	0.9732
Sb								1	0.0411	0.0995
Co									1	0.9021
V										1

**Table 5b:** Results from Spearman correlation.

Variables	Cu	Pb	Cr	As	Zn	Cd	Ni	Sb	Co	V
Cu	1	0.8424	0.9030	0.8511	0.1879	0.3421	0.9152	0.1159	0.9442	0.9573
Pb		1	0.8303	0.6991	0.0061	0.1774	0.8667	− 0.2378	0.8817	0.9086
Cr			1	0.7842	0.1273	0.1330	0.9758	0.0610	0.8754	0.9269
As				1	0.1885	0.0350	0.7173	− 0.1315	0.7997	0.8318
Zn					1	0.1267	0.1394	0.6525	0.2001	− 0.0061
Cd						1	0.2091	0.4494	0.2321	0.2390
Ni							1	0.1342	0.8879	0.9451
Sb								1	0.0126	− 0.0491
Co									1	0.9185
V										1

**Table 5c:** Results from Kendall correlation.

Variables	Cu	Pb	Cr	As	Zn	Cd	Ni	Sb	Co	V
Cu	1	0.7333	0.7778	0.6742	0.0667	0.2981	0.7778	0.1137	0.8355	0.8866
Pb		1	0.6889	0.5843	− 0.0222	0.1491	0.6889	− 0.1137	0.7400	0.7957
Cr			1	0.6293	0.1111	0.0994	0.9111	0.0682	0.6922	0.8411
As				1	0.1348	0.0251	0.5394	− 0.9196	0.6739	0.6897
Zn					1	0.0994	0.1111	0.5229	0.1194	− 0.0227
Cd						1	0.1491	0.2796	0.2402	0.1779
Ni							1	0.1137	0.7400	0.8866
Sb								1	0.0244	0.0000
Co									1	0.7814
V										1

**Table 6a:** Results of transformation 1.

Variables	Cu	Pb	Cr	As	Zn	Cd	Ni	Sb	Co	V
Cu	0	0.2512	0.0551	0.0719	0.0025	0.0623	0.0356	0.0394	0.0034	0.0065
Pb		0	0.1939	0.1341	0.0760	0.1863	0.0861	0.0450	0.1448	0.2339
Cr			0	0.0386	0.1554	0.4618	0.0824	0.0357	0.1208	0.0235
As				0	0.0518	0.2034	0.1529	0.0426	0.0895	0.0504
Zn					0	0.3082	0.0454	0.4356	0.0613	0.0432
Cd						0	0.2305	0.0209	0.1475	0.2349
Ni							0	0.0856	0.0718	0.0280
Sb								0	0.0285	0.1486
Co									0	0.0165
V										0

**Table 6b:** Results of transformation 2.

Variables	Cu	Pb	Cr	As	Zn	Cd	Ni	Sb	Co	V
Cu	0	0.1421	0.0701	0.2488	0.1187	0.1063	0.1730	0.0416	0.1122	0.0772
Pb		0	0.0525	0.0194	0.1043	0.2146	0.0917	0.1692	0.0031	0.1210
Cr			0	0.1164	0.1392	0.4954	0.0178	0.0285	0.0624	0.1092
As				0	0.0018	0.2133	0.3309	0.0031	0.2133	0.1925
Zn					0	0.3355	0.0171	0.3061	0.1421	0.0599
Cd						0	0.2905	0.1489	0.1394	0.2960
Ni							0	0.0652	0.2197	0.0866
Sb								0	0.0167	0.0995
Co									0	0.1207
V										0

**Table 6c:** Results of transformation 3.

Variables	Cu	Pb	Cr	As	Zn	Cd	Ni	Sb	Co	V
Cu	0	0.1091	0.1253	0.1769	0.1212	0.0440	0.1374	0.0022	0.1087	0.0707
Pb		0	0.1414	0.1148	0.0283	0.0283	0.1778	0.1241	0.1417	0.0113
Cr			0	0.1550	0.0162	0.0337	0.0647	0.0072	0.1832	0.0857
As				0	0.0536	0.0098	0.1780	0.0395	0.1238	0.1421
Zn					0	0.0273	0.0283	0.1296	0.0808	0.0166
Cd						0	0.0600	0.1698	0.0081	0.0611
Ni							0	0.0205	0.1479	0.0586
Sb								0	0.0118	0.0491
Co									0	0.1371
V										0
